# A systematic review of cost-effectiveness analyses of complex wound interventions reveals optimal treatments for specific wound types

**DOI:** 10.1186/s12916-015-0326-3

**Published:** 2015-04-22

**Authors:** Andrea C Tricco, Elise Cogo, Wanrudee Isaranuwatchai, Paul A Khan, Geetha Sanmugalingham, Jesmin Antony, Jeffrey S Hoch, Sharon E Straus

**Affiliations:** Knowledge Translation Program, Li Ka Shing Knowledge Institute, St. Michael’s Hospital, 30 Bond Street, Toronto, ON M5B 1W8 Canada; Epidemiology Division, Dalla Lana School of Public Health, University of Toronto, 155 College Street, Toronto, ON M5T 3M7 Canada; Institute of Health Policy, Management and Evaluation, University of Toronto, 155 College Street, Toronto, ON M5T 3M7 Canada; Department of Geriatric Medicine, University of Toronto, 200 Elizabeth Street, Suite RFE 3-805, Toronto, ON M5G 2C4 Canada

**Keywords:** Complex wound, Cost-benefit analysis, Cost-effectiveness analysis, Research design, Skin ulcer, Systematic review

## Abstract

**Background:**

Complex wounds present a substantial economic burden on healthcare systems, costing billions of dollars annually in North America alone. The prevalence of complex wounds is a significant patient and societal healthcare concern and cost-effective wound care management remains unclear. This article summarizes the cost-effectiveness of interventions for complex wound care through a systematic review of the evidence base.

**Methods:**

We searched multiple databases (MEDLINE, EMBASE, Cochrane Library) for cost-effectiveness studies that examined adults treated for complex wounds. Two reviewers independently screened the literature, abstracted data from full-text articles, and assessed methodological quality using the Drummond 10-item methodological quality tool. Incremental cost-effectiveness ratios were reported, or, if not reported, calculated and converted to United States Dollars for the year 2013.

**Results:**

Overall, 59 cost-effectiveness analyses were included; 71% (42 out of 59) of the included studies scored 8 or more points on the Drummond 10-item checklist tool. Based on these, 22 interventions were found to be more effective and less costly (i.e., dominant) compared to the study comparators: 9 for diabetic ulcers, 8 for venous ulcers, 3 for pressure ulcers, 1 for mixed venous and venous/arterial ulcers, and 1 for mixed complex wound types.

**Conclusions:**

Our results can be used by decision-makers in maximizing the deployment of clinically effective and resource efficient wound care interventions. Our analysis also highlights specific treatments that are not cost-effective, thereby indicating areas of resource savings.

Please see related article: http://dx.doi.org/10.1186/s12916-015-0288-5

**Electronic supplementary material:**

The online version of this article (doi:10.1186/s12916-015-0326-3) contains supplementary material, which is available to authorized users.

## Background

Complex wounds are those that do not heal after a period of 3 months or more [1]. These types of wounds are a significant burden on the healthcare system and result in patient and caregiver stress, economic loss, and decreased quality of life. At least 1% of individuals living in high economy countries will experience a complex wound in their lifetime [2], and over 6.5 million individuals have a complex wound in the United States alone [3]. Moreover, these types of wounds have a significant economic impact. For example, $10 billion United States dollars (USD) per year in North America is spent managing complex wounds [4], and 4% of the annual National Health Service expenditure in the United Kingdom is spent on care for patients with pressure ulcers [5].

There are three main categories of complex wounds: i) wounds resulting from chronic disease (e.g., venous insufficiency, diabetes), ii) pressure ulcers, and iii) non-healing surgical wounds [6-8]. Treatment is targeted to the type of wound. Managing complex wounds resulting from disease usually involves improving the underlying disease; for example, optimizing diabetes control for patients with diabetes [9]. A clinical assessment and history of mobility and neurological disability is often necessary to treat patients with pressure ulcers [9]. Considerations for managing surgical wound infections include previous antibiotic treatment and immune response [3].

It is estimated that the global wound care market will reach over $22 billion USD annually by 2020 [10]. Due to the burgeoning costs from the management of patients requiring complex wound care, policymakers are interested in finding cost-effective treatments. However, the cost-effectiveness of all interventions available to treat complex wounds is currently unclear. As such, we sought to elucidate cost-effective treatment strategies for complex wounds through a systematic review of cost-effectiveness analyses.

## Methods

### Protocol

The systematic review question was posed by members of the Toronto Central Local Health Integrated Network. In collaboration with the Toronto Central Local Health Integrated Network, our research team prepared a draft protocol that was revised to incorporate feedback from systematic review methodologists, policymakers, and clinicians with expertise in wound care (Additional file 1). Our protocol also included conducting a related project comprising an overview of systematic reviews for treating complex wounds, and these results are available in a separate publication [11].

### Information sources and search strategy

On October 26, 2012, an experienced librarian conducted comprehensive literature searches in the following electronic databases from inception onwards: MEDLINE, EMBASE, and the Cochrane Library. The literature search was limited to adult patients and economic studies. The Peer Review of Electronic Search Strategies (PRESS) checklist [12] was used by another expert librarian to peer review the literature search. The search was revised, as necessary, and the final MEDLINE search is presented in Additional file 2. Full literature searches for the other databases are available upon request. The reference lists of the included studies were searched to identify additional relevant studies.

### Eligibility criteria

Inclusion criteria were defined using the ‘Patients, interventions, comparators, outcomes, study designs, timeframe’ (PICOST) framework [13], as follows:

#### Patients

Adults aged 18 years and older experiencing complex wounds. Complex wounds included those due to chronic disease (such as diabetic foot ulcers or venous leg ulcers), pressure ulcers (such as decubitus ulcers or bed sores), and non-healing surgical wounds.

#### Interventions

All complex wound care interventions were included, as identified from our overview of systematic reviews [11] and outlined in Additional file 3.

#### Comparators

All comparators were eligible for inclusion, including any of the eligible interventions in comparison with each other or versus no treatment or placebo or usual care.

#### Outcomes

Cost-effectiveness (i.e., both incremental cost and incremental effectiveness) was included, where effectiveness was measured by at least one of the following outcomes: quality-adjusted life-years (QALYs), wounds healed, ulcer-free/healing time, wound size reduction/improvement, or hospitalizations (number/length of stay).

#### Study designs

Economic evaluations were included in which the incremental cost-effectiveness ratios (ICERs) were reported or could be derived.

#### Timeframe

We did not limit inclusion to year of publication.

#### Other limitations

We limited cost-effectiveness analyses to those based on a study with a control group, and where the data were from direct comparisons (versus a review using indirect data). Both published and unpublished studies were eligible for inclusion. Although we focused inclusion on those studies written in English, we contacted the authors of potentially relevant non-English studies to obtain the English translation.

### Screening process for study selection

The team pilot-tested the pre-defined eligibility criteria using a random sample of 50 included titles and abstracts. After 90% agreement was reached, each title and abstract was screened by two team members, independently, using our Synthesi.SR tool [14]. Discrepancies were resolved by discussion or the involvement of a third reviewer. The same process was followed for screening full-text articles that were identified as being potentially relevant after screening their titles and abstracts.

### Data abstraction and data collection process

The team pilot-tested data abstraction forms using a random sample of five included cost-effectiveness analyses. Subsequently, two investigators independently read each article and abstracted relevant data. Differences in abstraction were resolved by discussion or the involvement of a third reviewer. Data items included study characteristics (e.g., type of economic evaluation, time horizon, treatment interventions examined, study comparators), patient characteristics (e.g., clinical population, wound type), and cost-effectiveness results (e.g., ICERs, cost per QALY, cost per wound healed). The perspective of the economic evaluation was categorized as: patient, public payer, provider, healthcare system, or society [15].

Cost-effectiveness studies can have four possible overall results, which are often represented graphically in quadrants on a cost-effectiveness plane [16]. The possibilities for the intervention versus a comparator are: 1) more effective and less costly, which we noted as ‘dominant’; 2) more effective and more costly; 3) less effective and less costly; and 4) less effective and more costly, which we noted as ‘dominated’. The first possibility is considered to be cost-effective; whereas possibility 4 is not cost-effective. Situations 2 and 3 requires judgment by the decision-maker to interpret [17], and in such cases, the decision is often dependent on the decision-maker’s willingness to pay. For interventions that were found to be more effective yet more costly (i.e., situation 2) or less effective and less costly (situation 3), ICERs were reported or derived from both the differences in cost (i.e., incremental cost) and effectiveness (i.e., incremental effectiveness) between the study’s intervention and comparator groups using the formula:

(Cost of the intervention – Cost of the comparator) ÷ (Effectiveness of the intervention – Effectiveness of the comparator)

To assess key variables influencing the cost-effectiveness results, sensitivity analyses, level of uncertainty in the cost and benefit estimates, and incremental variabilities (i.e., the variability of the incremental cost and the variability of the incremental effectiveness), were reported.

Authors of the included cost-effectiveness analyses were contacted for data verification, as necessary. Further, multiple studies reporting the same economic data were sorted into the major publication (e.g., most recent paper or largest sample size) and companion report. Our results focus on the major publications and the companion reports were used to provide supplementary material.

### Methodological quality appraisal

The methodological quality of the cost-effectiveness analyses was appraised using a 10-item tool developed by Drummond et al. (Additional file 4) [18]. The items on this tool include the appraisal of question definition, description of competing alternatives, effectiveness of the intervention, consideration of all relevant costs, measurement of costs, valuation of costs and consequences, cost adjustment/discounting, incremental analysis, uncertainty/sensitivity analysis, and discussion of study results. The Drummond score can range from 0 to 10. Each included cost-effectiveness analysis was appraised by two team members and conflicts were resolved by discussion or the involvement of a third reviewer.

### Synthesis

Since the purpose of this systematic review was to summarize the cost-effectiveness of interventions for complex wound care, the results are reported descriptively. The costing data from all studies were converted to 2013 USD to increase the comparability of the economic results across cost-effectiveness studies. This process entailed first converting the currencies into USD using purchasing power parities for the particular year of the data [19,20], and then adjusting these for inflation to the year 2013 (rounded to the nearest dollar) using the consumer price index for medical care in the United States [21].

## Results

### Literature search and screening

**Figure 1:**
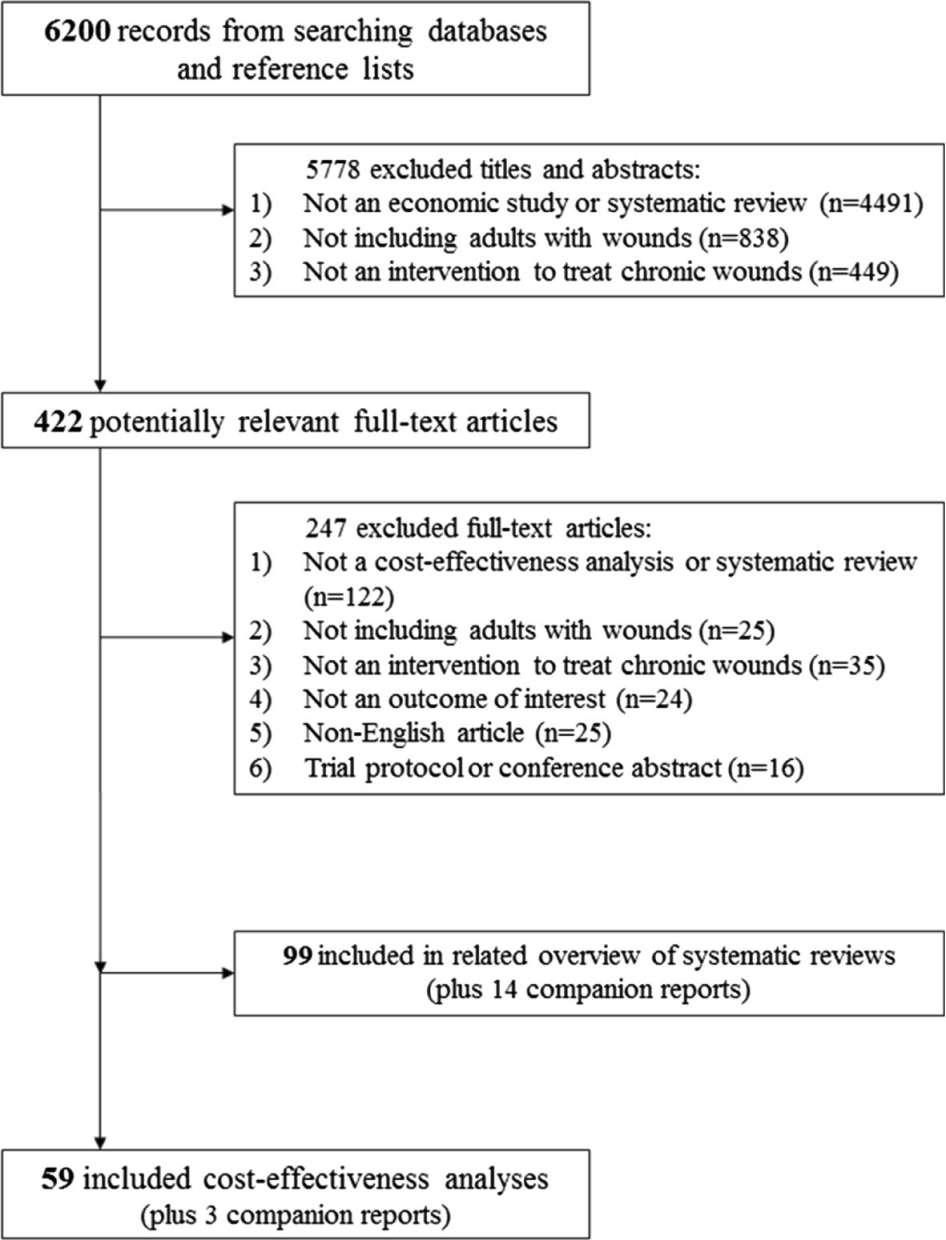
**Study flow diagram.**

The literature search identified 422 potentially relevant full-text articles after screening 6,200 titles and abstracts (Figure 1). There were 59 included cost-effectiveness analyses that fulfilled our eligibility criteria and were included [22-80], plus an additional three companion reports [81-83].

### Study and patient characteristics

The cost-effectiveness analyses evaluated interventions to treat venous ulcers (41%), diabetic ulcers (27%), and pressure ulcers (24%) (Table 1). The studies were published between 1988 and 2012. Most of the papers were conducted in the United Kingdom (29%) and United States (27%). Almost half (49%) reported private or mixed (private and public) funding sources of the studies, while one-third (34%) did not report a source of funding.

**Table 1:** **Summary characteristics of all cost-effectiveness analyses (CEAs)**

**Characteristic**	**No. of CEAs (n = 59)**	**Percentage of CEAs**
**Original year of values**		
1982–1996	15	25.4
1997–2000	19	32.2
2001–2005	10	16.9
2006–2010	15	25.4
**Year of publication**		
1988–1996	7	11.9
1997–2001	21	35.6
2002–2006	12	20.3
2007–2012	19	32.2
**Country of conduct**		
Europe (17 from the UK)	34	57.6
North America (16 from USA)	19	32.2
Asia	3	5.1
Australia and New Zealand	3	5.1
**Perspective**		
Public payer	17	28.8
Society	8	13.6
Provider	6	10.2
Health care system	1	1.7
Not reported	27	45.8
**Efficacy study design**		
RCT	44	74.6
Observational	9	15.3
Systematic review of RCT	4	6.8
Systematic review^a^	1	1.7
Pseudo-RCT	1	1.7
**Sample size** ^**b**^		
10–30	4	6.8
31–50	11	18.6
51–100	12	20.3
101–150	5	8.5
151–200	3	5.1
201–400	16	27.1
>400	8	13.6
**Patient age** ^**c**^ **(years)**		
50–59	5	8.5
60–69	20	33.9
70–79	18	30.5
80–89	8	13.6
Not reported	8	13.6
**Timeframe**		
≤12 weeks	28	47.5
13–24 weeks	9	15.3
>24 weeks	22	37.3
**Funding source** ^**d**^		
Private	23	39.0
Public	10	16.9
Mixed	6	10.2
Not reported	20	33.9
**Type of wound**		
Venous ulcers	24	40.7
Diabetic ulcers	16	27.1
Pressure ulcers	14	23.7
Mixed wounds	3	5.1
Mixed venous and venous/arterial ulcers	2	3.4
**Unit of effectiveness**		
Additional wound healed	26	44.1
QALY gained	10	16.9
Ulcer-free time (day/week/month) gained	9	15.3
Percentage additional reduction of ulcer (area/volume/volume per week)	8	13.6
Increase in healing rate	2	3.4
Reduction in DESIGN score	1	1.7
Patient-year gained	1	1.7
Hospital-free day gained	1	1.7
Foot-related hospitalization avoided	1	1.7
**Interventions** ^**e**^		
Dressings	17	24.3
Bandage	12	17.1
Biologics	8	11.4
Topical Tx	8	11.4
Wound care programs	7	10.0
Devices	5	7.1
Skin replacement Tx	4	5.7
Oral Tx	3	4.3
Support surfaces	2	2.9
Stockings	1	1.4
Surgery	1	1.4
Wound cleansing	1	1.4
Unspecified	1	1.4
**Comparators** ^**e**^		
Dressings	17	24.3
Bandage	8	11.4
No Tx	6	8.6
Biologics	4	5.7
Stockings	2	2.9
Support surfaces	2	2.9
Topical Tx	2	2.9
Wound care programs	2	2.9
Devices	1	1.4
Surgery	1	1.4
Usual care/Unspecified	25	35.7

QALY, Quality-adjusted life-year; RCT, Randomized clinical trial; Tx, Therapy/treatment.

^**a**^Not specified if the included studies were RCTs.

^**b**^For studies based on a review, this refers to the total sample size of the combined studies that the data were estimated from.

^**c**^Age here refers to mean age or the age used in the model.

^**d**^Mixed here indicates both private and public funding.

^**e**^Numbers do not add up to 59 as some studies contributed data to more than one category.

While the majority of studies based effectiveness on a (single) randomized clinical trial (75%), only a few based effectiveness on a systematic review (9%) and 15% were based on observational studies (Tables 2, 3, 4, 5 and 6). Almost half (46%) of the economic studies included a sample size of 10 to 100 patients and the rest had a sample of >100 patients. In addition, 48% were conducted in a timeframe of 12 weeks or less, while the other studies had a duration of >12 weeks follow-up. Across the 59 economic studies, 9 different units of effectiveness were used, with the most common ones being healed wound (44%) and QALY (17%). Regarding the perspective of the cost-effectiveness analysis, almost half (46%) did not report this explicitly and 29% reported using the public payer perspective.

**Table 2:** **Characteristics of each cost-effectiveness analysis (CEA) for venous ulcers (n = 24)**

**CEA (Original year of values)**	**Country (Original currency)**	**Perspective**	**Efficacy study design**	**Sample size**	**Population**	**Timeframe**	**Funding source** ^**a**^
Augustin 1999 (1989) [22]	Germany (DM)	Not reported	RCT	25	Mean 61 yrs; venous insufficiency	24 wks	Not reported
DePalma 1999 (1998) [23]	USA (US$)	Not reported	RCT	38	Mean 61 yrs; venous insufficiency	max. 12 wks	Private
Glinski 1999 (1998) [24]	Poland (PLN)	Public payer	RCT	140	Mean 65 yrs; venous insufficiency	24 wks	Not reported
Gordon 2006 (2005) [25]	Australia (AU$)	Society	RCT	56	Most >71 yrs; venous insufficiency	24 wks	Not reported
Guest 2012 (2010) [26]	UK (£)	Public payer	Observational	510	Mean 80 yrs; venous insufficiency	24 wks	Private
Iglesias 2006 (2004) [27]	UK (£)	Public payer	SR of RCTs	434	66 yrs; venous insufficiency	52 wks	Public
Iglesias 2004 (2001) [28]	UK (£)	Public payer	RCT	387	Mean 71 yrs; venous insufficiency	52 wks	Public
Jull 2008 (2005) [29]	New Zealand (NZ$)	Public payer	RCT	368	Mean 68 yrs; venous insufficiency	12 wks	Mixed
Junger 2008 (2007) [30]	Germany (DM)	Not reported	RCT	39	Mean 67 yrs; venous insufficiency	17 wks	Private
Kerstein 2000 (1995) [31]	USA (US$)	Not reported	Observational	81	Mean 65 yrs; venous insufficiency	3 yrs	Not reported
Kikta 1988 (1987) [32]	USA (US$)	Not reported	RCT	87	Venous insufficiency; (ages NR)	24 wks	Not reported
Michaels 2009 (2007) [33]	UK(£)	Public payer	RCT	213	Mean 71 yrs; venous insufficiency	12 wks	Public
Morrell 1998 (1995) [34]	UK (£)	Public payer	RCT	233	Mean 74 yrs; venous insufficiency	52 wks	Public
O’Brien 2003 (2000) [35]	Ireland (€)	Public payer	RCT	200	Mean 72 yrs; venous insufficiency	12 wks	Private
Oien 2001 (1997) [36]	Sweden (£)	Not reported	Observational	68	Mean 76 yrs; venous insufficiency	12 wks	Not reported
Sibbald 2001 (1997) [37]	Canada (CAN$)	Society	RCT	293	Elderly; venous insufficiency	13 wks	Private
Taylor 1998 (1987) [38]	UK (£)	Not reported	RCT	36	Mean 75 yrs; venous insufficiency	12 wks	Private
Ukat 2003 (2002) [39]	Germany (€)	Not reported	RCT	89	Mean 69 yrs; venous insufficiency	12 wks	Private
Watson 2011 (2007) [40]	UK (£)	Public payer	RCT	337	Mean 69 yrs; venous insufficiency	52 wks	Public
Pham 2012 (2009) [41]	Canada (CAN$)	Society	RCT	424	Mean 65 yrs; venous insufficiency; most fully mobile	max. 52 wks	Public
Schonfeld 2000 (1996) [42]	USA(US$)	Public payer	RCT	240	Mean 60 yrs; venous insufficiency	52 wks	Private
Simon 1996 (1993) [43]	UK (£)	Not reported	Observational	901	Venous insufficiency; (ages not reported)	13 wks	Mixed
Carr 1999 (1998) [44]	UK (£)	Public payer	RCT	233	Mean 73 yrs; venous insufficiency	52 wks	Private
Guest 2009 (2007) [45]	UK (£)	Public payer	RCT	83	Mean 71 yrs; venous insufficiency	52 wks	Private

RCT, Randomized clinical trial; SR, Systematic review; wks, Weeks; yrs, Years.

^**a**^Mixed here indicates both private and public funding.

**Table 3:** **Characteristics of each cost-effectiveness analysis (CEA) for venous and venous/arterial ulcers (n = 2)**

**CEA (Original year of values)**	**Country (Original currency)**	**Perspective**	**Efficacy study design**	**Sample size**	**Population**	**Timeframe**	**Funding source**
Dumville 2009 (2006) [46]	UK (£)	Public payer	RCT	267	Mean 74 yrs; venous insufficiency	52 wks	Not reported
Ohlsson 1994 (1993) [47]	Sweden (SEK)	Not reported	RCT	30	Median 76 yrs; venous insufficiency; most female	6 wks	Not reported

RCT, Randomized clinical trial; WKS, Weeks; Yrs, Years.

**Table 4:** **Characteristics of each cost-effectiveness analysis (CEA) for diabetic ulcers (n = 16)**

**CEA (Original year of values)**	**Country (Original currency)**	**Perspective**	**Efficacy study design**	**Sample size**	**Population**	**Timeframe**	**Funding source** ^**a**^
Abidia 2003 (2000) [48]	UK (£)	Not reported	RCT	18	Mean 71 yrs; diabetes	52 wks	Not reported
Apelqvist 1996 (1993) [49]	Sweden (SEK)	Society	RCT	41	Included >40 yrs; diabetes	12 wks	Mixed
Edmonds 1999 (1996) [50]	UK (£)	Provider	RCT	40	Mean 66 yrs; diabetes; foot infections	2 wks	Private
Guo 2003 (2001) [51]	USA (US$)	Society	SR^b^	126	60 yrs; diabetes	12 yrs	Not reported
Habacher 2007 (2001) [52]	Austria (€)	Society	Observational	119	Mean 65 yrs; diabetes	15 yrs	Not reported
Horswell 2003 (1999) [53]	USA (US$)	Not reported	Observational	214	Mean 54 yrs; diabetes; mostly African-Americans	52 wks	Not reported
Jansen 2009 (2006) [54]	UK (£)	Public payer	RCT	402	Mean 58 yrs; diabetes	approx. 4 wks	Private
Jeffcoate 2009 (2007) [55]	UK (£)	Public payer	RCT	317	Mean 60 yrs; diabetes	24 wks	Public
McKinnon 1997 (1994) [56]	USA (US$)	Provider	RCT	90	Mean 60 yrs; diabetes; limb-threatening foot infections	3 wks	Private
Persson 2000 (1999) [57]	Sweden (US$)	Not reported	SR of RCTs	500	Median 60 yrs; diabetes	52 wks	Private
Piaggesi 2007 (2006) [58]	Italy (€)	Not reported	RCT	40	Mean 60 yrs; diabetes	12 wks	Private
Redekop 2003 (1999) [59]	The Nether-lands (€)	Society	RCT	208	Elderly; diabetes	52 wks	Private
Allenet 2000 (1998) [60]	France (FF)	Society	RCT	235	Diabetes; (ages not reported)	52 wks	Not reported
Ghatnekar 2002 (2000) [61]	France (€)	Not reported	RCT	157	Diabetes; (ages not reported)	52 wks	Private
Ghatnekar 2001 (1999) [62]	UK(US$)	Public payer	SR of RCTs	449	Diabetes; (ages not reported)	52 wks	Private
Hailey 2007 (2004) [63]	Canada (CAN$)	Public payer	SR of RCTs	305	65 yrs; diabetes	12 yrs	Public

RCT, Randomized clinical trial; SR, Systematic review; wks, Weeks; yrs, Years.

^**a**^Mixed here indicates both private and public funding.

^**b**^Not specified if the included studies were RCTs or not (but states they were prospective controlled clinical studies).

**Table 5:** **Characteristics of each cost-effectiveness analysis (CEA) for pressure ulcers (n = 14)**

**CEA (Original year of values)**	**Country (Original currency)**	**Perspective**	**Efficacy study design**	**Sample size**	**Population**	**Timeframe**	**Funding source** ^**a**^
Branom 2001 (2000) [64]	USA (US$)	Not reported	RCT	20	Mean 72 yrs; bedridden	max. 8 wks	Not reported
Burgos 2000 (1998) [65]	Spain (Pta)	Not reported	RCT	37	Mean 80 yrs	12 wks	Private
Chang 1998 (1997) [66]	Malaysia (RM)	Not reported	RCT	34	Mean 58 yrs	max. 8 wks	Private
Chuangsu-wanich 2011 (2010) [67]	Thailand (US$)	Not reported	RCT	45	Mean 66 yrs	8 wks	Not reported
Ferrell 1995 (1992) [68]	USA (US$)	Provider	RCT	84	Mean 81 yrs; mostly Caucasians; most fecal incontinence	52 wks	Mixed
Foglia 2012 (2010) [69]	Italy (€)	Provider	Observational	362	Most >80 yrs	4.3 wks	Not reported
Graumlich 2003 (2001) [70]	USA (US$)	Not reported	RCT	65	Mean 83 yrs	8 wks	Public
Muller 2001 (1998) [71]	The Netherlands (NLG)	Provider	RCT	24	Mean 73 yrs; all females	12 wks	Private
Narayanan 2005 (2004) [72]	USA (US$)	Not reported	Observational	976	Most ≥80 yrs; mostly Caucasians	approx. 22 wks	
Payne 2009 (2007) [73]	USA (US$)	Provider	RCT	36	Mean 73 yrs	4 wks	Private
Robson 2000 (1999) [74]	USA (US$)	Not reported	RCT	61	Mean 50 yrs; mostly Caucasians	5 wks	Mixed
Sanada 2010 (2007) [75]	Japan (Yen)	Not reported	Observational	105	Mean 75 yrs	3 wks	Not reported
Xakellis 1992 (1990) [76]	USA (US$)	Not reported	RCT	39	Mean 80 yrs	1.4 wks	Mixed
Seberrn 1986 (1985) [77]	USA (US$)	Not reported	RCT	77	Mean 74 yrs	8 wks	Not reported

RCT, Randomized clinical trial; SR, Systematic review; wks, Weeks; yrs, Years.

^**a**^Mixed here indicates both private and public funding.

**Table 6:** **Characteristics of each cost-effectiveness analysis (CEA) for mixed wound types (n = 3)**

**CEA (Original year of values)**	**Country (Original currency)**	**Perspective**	**Efficacy study design**	**Sample size**	**Population**	**Timeframe**	**Funding source**
Bale 1998 (1994) [78]	UK (£)	Not reported	RCT	100	Mean 76 yrs	max. 8 wks	Private
Terry 2009 (2008) [79]	USA (US$)	Not reported	RCT	160	Mean 58 yrs	6 wks	Public
Vu 2007 (2000) [80]	Australia (AU$)	Health care system	Pseudo-RCT	342	Mean 83 yrs	20 wks	Public

RCT, Randomized clinical trial; wks, Weeks; Yrs, Year.

### Methodological quality appraisal

Approximately 71% (42 out of 59) of the cost-effectiveness analyses had a score of 8 or higher out of a total possible score of 10 (Additional file 5, Figure 2). Using the Drummond 10-item tool [18], the key methodological shortcoming across the cost-effectiveness analyses was that only 51% (30 out of 59) had established the ‘effectiveness’ of the intervention using data from efficacy studies (i.e., systematic reviews, randomized clinical trials or observational studies) that had sufficiently large sample sizes according to the International Conference on Harmonisation guidelines for establishing efficacy [84]. Consistent methodological strengths across the cost-effectiveness analyses included a clear research question, costs and consequences measured in appropriate physical units, credibly valued costs and consequences, and discounted costs (when applicable).

**Figure 2:**
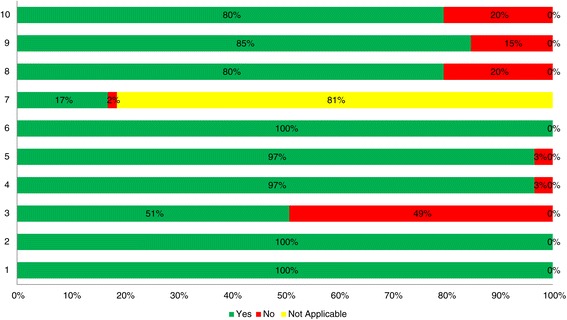
**Drummond methodological quality summary results (n = 59).** Items: **1.** Well-defined question. **2.** Competing alternatives well described. **3.** Effectiveness established. **4.** All important and relevant costs and consequences identified. **5.** Measurement accurately performed. **6.** Valuation credibility. **7.** Discounting. **8.** Incremental analysis performed. **9.** Allowance made for uncertainty. **10.** Discussion.

### Cost-effectiveness results

Due to the large number of cost-effectiveness studies included and the numerous results, we have focused on dominant results in the text. However, all of the cost-effectiveness results are presented in Tables 7, 8, 9, 10 and 11 and the sensitivity analyses, level of uncertainty, and incremental variabilities are outlined in Additional file 6.

**Table 7:** **Cost-effectiveness analysis (CEA) outcomes for venous ulcers (n = 24)**

**CEA (Original year of values)**	**Treatment vs. Comparator**	**ICER summary/estimate [2013 US$]**	**Unit of effectiveness**	**Incremental cost [2013 US$]**	**Incremental effectiveness**
Augustin 1999 (1989) [22]	Hydrocolloid dressing vs. Vaseline gauze dressing	Dominant	Ulcer-free week gained	−3,362	1.3
DePalma 1999 (1998) [23]	Thera-boot vs. Unna’s boot	Dominant	Ulcer-free week gained	−601	1.71
Glinski 1999 (1998) [24]	Micronized purified flavonoid fraction + SC vs. SC alone	Dominant^a^	Additional wound healed	−714	0.19
Gordon 2006 (2005) [25]	Community leg club vs. community home nursing	488^a^	Additional wound healed	Not reported	Not reported
Guest 2012^b^ (2010) [26]	NSBF vs. DBC	18^a^	Percent additional reduction of ulcer area	146	8
Guest 2012^b^ (2010) [26]	NSBF vs. no skin protectant	1^a^	Percent additional reduction of ulcer area	17	22
Guest 2012^b^ (2010) [26]	DBC vs. no skin protectant	Dominant^a^	Percent additional reduction of ulcer area	−129	14
Iglesias 2006 (2004) [27]	Pentoxifylline plus compression vs. placebo plus compression	Dominant^a^	QALY gained	−213	0.01
Iglesias 2004 (2001) [28]	Four-layer bandage vs. short-stretch bandage	Dominant^a^	QALY gained	−566	0.02
Jull 2008 (2005) [29]	Manuka honey dressing vs. UC	Dominant^a,c^	Additional wound healed	−48	0.06
Junger 2008 (2007) [30]	Low-frequency pulsed current (Dermapulse) vs. placebo	More costly & more effective^d^	Percent additional reduction of ulcer area	Not reported	Not reported
Kerstein 2000^b^ (1995) [31]	Hydrocolloid dressing plus compression hosiery vs. Unna’s boot	Dominant	Additional wound healed	−6,748	0.18
Kerstein 2000^b^ (1995) [31]	Unna’s boot vs. saline gauze plus compression hosiery	More costly & more effective^d^	Additional wound healed	Not reported	Not reported
Kikta 1988 (1987) [32]	Unna’s boot vs. hydrocolloid (DuoDERM)	Dominant^a^	Additional wound healed	−209	0.32
Michaels 2009 (2007) [33]	Antimicrobial silver-donating dressings vs. low-adherent dressings	917,298^a^	QALY gained	183	0.0002
Morrell 1998 (1995) [34]	Community leg ulcer clinics using four-layer compression bandaging vs. home nursing UC	7^a^	Ulcer-free week gained	44	5.9
O’Brien 2003 (2000) [35]	Four-layer bandage vs. UC	Dominant^a^	Increase in healing rate	−42	0.2
Oien 2001 (1997) [36]	Pinch grafting in primary care vs. pinch grafting in hospital	Cost saving & same effectiveness	Additional wound healed	−14,075	0
Sibbald 2001 (1997) [37]	Skin substitute (Apligraf) plus four-layer bandage vs. four-layer bandage only	6095^a^	Additional wound healed	457	0.075
Taylor 1998 (1987) [38]	Four-layer high-compression bandaging vs. UC	Dominant^a^	Additional wound healed	−659	0.095
Ukat 2003 (2002) [39]	Multilayer elastic bandaging (Profore) vs. short-stretch bandaging	Dominant^a^	Additional wound healed	−1,198	0.08
Watson 2011 (2007) [40]	Ultrasound plus SC vs. SC alone	Dominated^a^	QALY gained	371	−0.009
Pham 2012 (2009) [41]	Four-layer bandaging vs. short-stretch bandaging	43,918^a^	QALY gained	395	0.009
Schonfeld 2000 (1996) [42]	Apligraf (Graftskin) vs. Unna’s Boot	Dominant^a^	Ulcer-free month gained	−13,883	2.85
Simon 1996 (1993) [43]	Community leg ulcer clinic vs. UC clinic	Dominant	Additional wound healed	−1,826	0.22
Carr 1999 (1998) [44]	Four-layer compression bandaging (Profore) vs. UC	Dominant^a^	Additional wound healed	−1,289	0.13
Guest 2009 (2007) [45]	Amelogenin plus compression therapy vs. compression therapy only	Dominant^a^	QALY gained	−835	0.054

DBC, Durable barrier cream; ICER, Incremental cost-effectiveness ratio; NSBF, No sting barrier film; QALY, Quality-adjusted life-year; SC, Standard care; UC, Usual care; US$, United States dollars.

^**a**^Denotes the higher quality studies (Drummond score ≥8).

^**b**^Multiple comparisons are reported.

^**c**^ICER was mostly due to an extra 3 patients hospitalized in control group… “probably due to random variation”. If remove these costs, the dominance is reversed in favor of UC.

^**d**^Unable to calculate specific ICER for these 2 studies because the data was not reported for all treatment arms or presented in a figure only but the overall result (more costly & more effective) was reported.

**Table 8:** **Cost-effectiveness analysis (CEA) outcomes for venous and venous/arterial ulcers (n = 2)**

**CEA (Original year of values)**	**Treatment vs. Comparator**	**ICER summary/estimate [2013 US$]**	**Unit of effectiveness**	**Incremental cost [2013 US$]**	**Incremental effectiveness**
Dumville 2009 (2006) [46]	larval therapy vs. hydrogel	17,757^a^	QALY gained	195	0.011
Ohlsson 1994 (1993) [47]	hydrocolloid (DuoDERM) dressing vs. saline gauze	Dominant^a^	Additional wound healed	−588	0.357

ICER, Incremental cost-effectiveness ratio; QALY, Quality-adjusted life-year; US$, United States dollars.

^**a**^Denotes the higher quality studies (Drummond score ≥8).

**Table 9:** **Cost-effectiveness analysis (CEA) outcomes for diabetic ulcers (n = 16)**

**CEA (Original year of values)**	**Treatment vs. Comparator**	**ICER summary/estimate [2013 US$]**	**Unit of effectiveness**	**Incremental cost [2013 US$]**	**Incremental effectiveness**
Abidia 2003 (2000) [48]	HBOT vs. control	Dominant	Additional wound healed	−7,596	0.625
Apelqvist 1996 (1993) [49]	Cadexomer iodine ointment vs. standard treatment	Dominant^a^	Additional wound healed	−119	0.183
Edmonds 1999 (1996) [50]	Filgrastim vs. placebo	Dominant^a,b^	Hospital-free day gained	−7,738	7.5
Guo 2003 (2001) [51]	HBOT + SC vs. SC alone	3508^a^	QALY gained	2,137	0.609
Habacher 2007 (2001) [52]	Intensified treatment vs. SC	Dominant^a^	Patient-year gained	−7,625	2.97
Horswell 2003 (1999) [53]	Staged management diabetes foot program vs. SC	Dominant^a^	Foot-related hospitalization avoided	−7,848	0.41
Jansen 2009 (2006) [54]	Ertapenem vs. Piperacillin/Tazobactam	Dominant^a^	Lifetime QALY gained	−822	0.12
Jeffcoate 2009^c^ (2007) [55]	Hydrocolloid (Aquacel) vs. antiseptic (Inadine)	1449^a^	Additional wound healed	14	0.01
Jeffcoate 2009^c^ (2007) [55]	Antiseptic (Inadine) vs. non-adherent dressing	1590^a^	Additional wound healed	80	0.05
McKinnon 1997 (1994) [56]	Ampicillin/sulbactam vs. imipenem/cilastatin	Dominant^a^	Hospitalization day avoided	−5,891	3.5
Persson 2000 (1999) [57]	Becaplermin plus GWC (unspecified) vs. GWC alone	Dominant^a^	Ulcer-free month gained	−628	0.81
Piaggesi 2007 (2006) [58]	Total contact casting vs. Optima Diab device	8,578	Additional wound healed	858	0.1
Redekop 2003 (1999) [59]	Apligraf (skin substitute) + GWC^d^ vs. GWC alone	Dominant^a^	Ulcer-free month gained	−1,223	1.53
Allenet 2000 (1998) [60]	Dermagraft (human dermal replacement) vs. SC	70,961^a^	Additional wound healed	12,652	0.178
Ghatnekar 2002 (2000) [61]	Promogran dressing plus GWC^e^ vs. GWC alone	Dominant^a^	Additional wound healed	−294	0.042
Ghatnekar 2001 (1999) [62]	Becaplermin gel (containing recombinant human platelet-derived growth factor) plus GWC^f^ vs. GWC alone	Dominant^a^	Ulcer-free month gained	−794	0.81
Hailey 2007 (2004) [63]	HBOT + SC vs. SC alone	Dominant	QALY gained	−9,337	0.63

GWC, Good wound care; HBOT, Hyperbaric oxygen therapy; ICER, Incremental cost-effectiveness ratio; QALY, Quality-adjusted life-year; SC, Standard care; US$, United States dollars.

^**a**^Denotes the higher quality studies (Drummond score ≥8).

^**b**^“Patient selection may have occurred during the in-hospital stay where more control patients experienced a bad vascular condition requiring the more costly interventions”.

^**c**^Multiple comparisons are reported.

^**d**^GWC, “the best wound care available and consists mainly of offloading, debridement, and moist dressings”.

^**e**^GWC, “sharp debridement (if necessary) and wound cleansing. In the GWC alone arm, the primary dressing was saline-soaked gauze and the secondary gauze and tape”.

^**f**^GWC, “sharp debridement to remove callus, fibrin and necrotic tissue; moist saline dressing changes every 12 hours; systematic control of infection, if present; glucose control; and offloading of pressure”.

**Table 10:** **Cost-effectiveness analysis (CEA) outcomes for pressure ulcers (n = 14)**

**CEA (Original year of values)**	**Treatment vs. Comparator**	**ICER summary/estimate [2013 US$]**	**Unit of effectiveness**	**Incremental cost [2013 US$]**	**Incremental effectiveness**
Branom 2001 (2000) [64]	Constant Force Technology mattress vs. low-air-loss mattress	Dominant	Percent additional reduction in wound volume per week	−1,435	0.04
Burgos 2000 (1998) [65]	Collagenase ointment vs. hydrocolloid (Varihesive) dressing	1,278	Percent additional reduction of ulcer area	20,825	16.3
Chang 1998 (1997) [66]	Hydrocolloid (DuoDERM CGF) vs. saline gauze	3	Percent additional reduction of ulcer area	121	43
Chuangsu-wanich 2011 (2010) [67]	Silver mesh dressing vs. silver sulfadiazine cream	Dominant	Increase in healing rate	−1,695	11.89
Ferrell 1995 (1992) [68]	Low-air-loss bed vs. conventional foam mattress	58^a^	Ulcer-free day gained	Not reported	Not reported
Foglia 2012 (2010) [69]	Advanced dressings vs. simple dressings	Dominant^a^	Percent additional reduction of ulcer area	−132	6
Graumlich 2003 (2001) [70]	Collagen (Medifil) vs. hydrocolloid (DuoDERM)	63,147^a^	Additional wound healed	632	0.01
Muller 2001 (1998) [71]	Collagenase-containing ointment (Novuxol) vs. hydrocolloid (DuoDERM) dressing	Dominant^a^	Additional wound healed	−149	0.281
Narayanan 2005^b^ (2004) [72]	Initial wound stage 1: BCT (balsam Peru + hydrogenated castor oil + trypsin ointment) only vs. BCT + Others (BCT plus Other treatments)	Dominant	Additional wound healed	−5	0.106
Narayanan 2005^b^ (2004) [72]	Initial wound stage 1: BCT + Others vs. Others	Dominant	Additional wound healed	−10	0.263
Narayanan 2005^b^ (2004) [72]	Initial wound stage 2: BCT only vs. Others	Dominant	Additional wound healed	−6	0.16
Narayanan 2005^b^ (2004) [72]	Initial wound stage 2: BCT only vs. BCT + Others	Dominant	Additional wound healed	−7	0.159
Narayanan 2005^b^ (2004) [72]	Initial wound stage 2: BCT + Others vs. Others	226,208	Additional wound healed	226	0.001
Payne 2009 (2007) [73]	Polyurethane foam dressing (Allevyn Thin) vs. saline gauze	Dominant	Additional wound healed	−564	0.181
Robson 2000^b^ (1999) [74]	Sequential GM-CSF and bFGF vs. bFGF only	Dominant	Percent additional reduction of ulcer volume	1,357	−0.07
Robson 2000^b^ (1999) [74]	Sequential GM-CSF and bFGF vs. GM-CSF only	Dominant	Percent additional reduction of ulcer volume	−848	1
Robson 2000^b^ (1999) [74]	Placebo vs. sequential GM-CSF and bFGF	735	Percent additional reduction of ulcer volume	2,205	3
Sanada 2010 (2007) [75]	New incentive system vs. non-introduced control	Dominant	reduction in DESIGN score	−16	4.1
Xakellis 1992 (1990) [76]	Hydrocolloid (DuoDERM) vs. gauze	Dominant^a^	ulcer-free day gained	−25	2
Sebern 1986^b^ (1985) [77]	Grade II PrU: MVP vs. gauze	Dominant^a^	percent additional reduction of ulcer area	−1,925	48
Sebern 1986^b^ (1985) [77]	Grade III PrU: MVP vs. gauze	9^a^	percent additional reduction of ulcer area	217	23

BCT, Balsam Peru plus hydrogenated castor oil plus trypsin ointment; bFGF, Basic fibroblast growth factor; GM-CSF, Granulocyte-macrophage/colony-stimulating factor; ICER, Incremental cost-effectiveness ratio; MVP, Moisture vapor permeable dressing; PrU, Pressure ulcer; QALY, Quality-adjusted life-year; US$, United States dollars.

^**a**^Denotes the higher quality studies (Drummond score ≥8).

^**b**^Multiple comparisons are reported.

**Table 11:** **Cost-effectiveness analysis (CEA) outcomes for mixed wound types (n = 3)**

**CEA (Original year of values)**	**Treatment vs. Comparator**	**ICER summary/estimate [2013 US$]**	**Unit of effectiveness**	**Incremental cost [2013 US$]**	**Incremental effectiveness**
Bale 1998 (1994) [78]	Hydrocellular (Allevyn) dressing vs. hydrocolloid (Granuflex) dressing	26	Additional wound healed	3	0.13
Terry 2009 (2008) [79]	Telemedicine plus WCS consults vs. WCS consults only	Dominated^a^	Additional wound healed	2,085	−0.249
Vu 2007 (2000) [80]	Multidisciplinary wound care team vs. UC	Dominant^b^	Additional wound healed	−346	0.092

ICER, Incremental cost-effectiveness ratio; UC, Usual care; US$, United States dollars; WCS, Wound care specialist.

^**a**^“Disproportionate distribution, by chance, in group A [telemedicine plus WCS consults] of large non-healing surgical wounds and large, numerous pressure ulcers”.

^**b**^Denotes the higher quality study (Drummond score ≥8).

#### Venous ulcers

Twenty-four cost-effectiveness analyses examined interventions for venous ulcers (Table 7) [22-45,83]. Sixteen studies found the interventions were dominant (i.e., more effective and less costly) [22-24,26-29,31,32,35,38,42-45], and 12 of these were studies with a Drummond score ≥8 [24,26-29,32,35,38,39,42,44,45]. These included Apligraf (Graftskin) vs. Unna’s Boot [42], Unna’s boot vs. hydrocolloid (DuoDERM) [32], micronized purified flavonoid fraction plus usual care vs. usual care alone [24], durable barrier cream vs. no skin protectant [26], pentoxifylline plus compression vs. placebo plus compression [27], Manuka honey dressing vs. usual care [29], amelogenin plus compression therapy vs. compression therapy only [45], and four-layer compression bandaging vs. usual care [35,38,44]. Although four-layer compression bandaging vs. short-stretch compression bandaging was found to be dominant in two studies [28,39]], this intervention was more effective and more costly in another economic evaluation [41].

Dominant interventions from four studies scoring <8 on the Drummond tool [22,23,31,43] included hydrocolloid dressing vs. Vaseline gauze dressing [22], hydrocolloid dressing plus compression hosiery vs. Unna’s boot [31], Thera-boot vs. Unna’s boot [23], and community leg ulcer clinic vs. usual care clinic [43].

#### Mixed venous and venous/arterial ulcers

Two cost-effectiveness analyses evaluated interventions for mixed venous and venous/arterial ulcers (Table 8) [46,47]. Only one study found an intervention to be dominant (and had a Drummond score ≥8); hydrocolloid (DuoDERM) dressing was dominant compared to saline gauze [47].

#### Diabetic ulcers

Sixteen cost-effectiveness analyses examined interventions for diabetic ulcers (Table 9) [48-63]. Twelve studies found the interventions were dominant [48-50,52-54,56,57,59,61-63], and 10 of these were studies with a Drummond score ≥8 [49,50,52-54,56,57,59,61,62]. These included becaplermin gel (containing recombinant human platelet-derived growth factor) plus good wound care (GWC) vs. GWC alone (note: the various GWC definitions used are outlined in Table 9) [57,62], cadexomer iodine ointment vs. usual care [49], filgrastim vs. placebo [50], intensified treatment vs. usual care [52], staged management diabetes foot program vs. usual care [53], ertapenem vs. piperacillin/tazobactam [54], ampicillin/sulbactam vs. imipenem/cilastatin [56], Apligraf (skin substitute) plus GWC vs. GWC alone [59], and promogran dressing plus GWC vs. GWC alone [61]. Hyperbaric oxygen therapy plus usual care vs. usual care alone was found to be dominant in one study [63], yet was more effective and more costly in another economic evaluation [51].

Dominant interventions from studies scoring <8 on the Drummond tool included hyperbaric oxygen therapy vs. control [48], and hyperbaric oxygen therapy plus standard care vs. standard care alone [63].

#### Pressure ulcers

Fourteen cost-effectiveness analyses evaluated pressure ulcer interventions (Table 10) [64-77]. Ten studies found the interventions were dominant [64,67,69,71-77], and four of these were studies with a Drummond score ≥8 [69,71,76,77]. These included moisture vapor permeable dressing vs. gauze [for grade II pressure ulcers] [77], advanced dressings vs. simple dressings [69], and hydrocolloid (DuoDERM) vs. gauze [76]. Collagenase-containing ointment (Novuxol) vs. hydrocolloid (DuoDERM) dressing was found to be dominant in one study [71], while collagen (Medifil) vs. hydrocolloid (DuoDERM) was more effective and more costly in another cost-effectiveness analysis [70].

The following interventions were dominant in six studies with a Drummond score <8: constant force technology mattress vs. low-air-loss mattress [64], silver mesh dressing vs. silver sulfadiazine cream [67], balsam Peru plus hydrogenated castor oil plus trypsin ointment vs. balsam Peru plus hydrogenated castor oil plus trypsin ointment plus other treatment (unspecified) for stage 1 and 2 wounds [72], balsam Peru plus hydrogenated castor oil plus trypsin ointment plus other treatment (unspecified) vs. other treatment (unspecified) for stage 1 wounds [72], balsam Peru plus hydrogenated castor oil plus trypsin ointment vs. other treatment (unspecified) for stage 2 wounds [72], polyurethane foam dressing vs. saline gauze [73], sequential granulocyte-macrophage/colony-stimulating factor and basic fibroblast growth factor vs. basic fibroblast growth factor alone [74], sequential granulocyte-macrophage/colony-stimulating factor and basic fibroblast growth factor vs. granulocyte-macrophage/colony-stimulating factor alone [74], and new hospital incentive system vs. non-introduced control [75].

#### Mixed wound types

Three cost-effectiveness analyses evaluated mixed complex wound types (Table 11) [78-80]. One study with a Drummond score ≥8 found that a multidisciplinary wound care team was dominant compared to usual care [80].

## Discussion

We conducted a comprehensive systematic review to summarize the cost-effectiveness of interventions for complex wound care including data from 59 cost-effectiveness analyses. These economic studies examined numerous interventions and comparators and used different outcomes to assess effectiveness. In a few situations, the intervention considered in one cost-effectiveness analysis comprised the comparator in another cost-effectiveness analysis. Therefore, cost-effectiveness results are presented as comparisons of one treatment option relative to another.

Based on evidence from 42 cost-effectiveness studies with a Drummond score ≥8, 22 intervention comparisons were dominant (Additional file 7). For venous ulcers, these were four-layer compression bandaging vs. usual care, skin replacement vs. Unna’s Boot, Unna’s boot vs. hydrocolloid, micronized purified flavonoid fraction plus usual care vs. usual care, durable barrier cream vs. no skin protectant, pentoxifylline plus compression vs. placebo plus compression, Manuka honey dressing vs. usual care, and amelogenin plus compression therapy vs. compression therapy only. For mixed venous and venous/arterial ulcers, only hydrocolloid dressing vs. saline gauze was dominant according to high quality cost-effectiveness analyses. For diabetic ulcers, cadexomer iodine ointment vs. usual care, filgrastim vs. placebo, intensified treatment vs. usual care, staged management diabetes foot program vs. usual care, ertapenem vs. piperacillin/tazobactam, ampicillin/sulbactam vs. imipenem/cilastatin, skin replacement plus GWC vs. GWC alone, promogran dressing plus GWC vs. GWC alone, and becaplermin gel (containing recombinant human platelet-derived growth factor) plus GWC vs. GWC alone were dominant. For pressure ulcers, moisture vapor permeable dressing vs. gauze, advanced dressings vs. simple dressings, and hydrocolloid vs. gauze were dominant. Finally, for mixed wound types, multidisciplinary wound care team was dominant vs. usual care.

Our results highlight a need for a future network meta-analysis given the numerous interventions and comparators available. Network meta-analysis is a statistical technique that can be used to combine direct evidence of effectiveness from head-to-head studies and indirect evidence of the relative benefits of interventions versus a common comparator (usually placebo). This powerful statistical approach can also be used to select the best treatment option available from a ranking of all treatments. An attractive property of network meta-analysis is that it allows researchers and health economists the opportunity to use the ranking analysis to generate a *de novo* cost-effectiveness analysis more efficiently. Another potential future study is to conduct a systematic review of clinical practice guidelines on complex wounds, and compare the interventions recommended in these with those found to be cost-effective in our review.

The major methodological quality limitation found in the included cost-effectiveness analyses was that the majority did not adequately establish the effectiveness of the wound care intervention using data from systematic reviews, randomized clinical trials, or observational studies that had sufficiently large sample sizes. Moreover, many of the included economic studies did not report on uncertainty of the cost-effectiveness estimates, incremental variabilities, or sensitivity analyses, thereby further limiting the utility of those results. Further, many of the cost-effectiveness analyses did not assess long-term cost-effectiveness, and the choice of timeframe for an economic evaluation might significantly affect the cost-effectiveness results. Given the chronic nature of many types of wounds, economic modeling of a longer time horizon would provide a clearer picture in many circumstances. As an example, an intervention might be more effective yet more costly in the first 2 months of usage but it might be cost saving over a 1 year or longer timeframe due to overall fewer additional interventions required. Furthermore, most of the cost-effectiveness studies did not include information on patient-reported quality of life, which is a major limitation of this literature.

The majority of the included economic studies were from European countries and 16 were from the United States. When trying to apply the cost-effectiveness results to a country-specific context, several factors need to be assessed such as the perspective of the economic evaluation (e.g., public payer, healthcare provider), the type of healthcare system (e.g., publicly-funded healthcare), the local practice of medicine, and local costs.

There are a few limitations related to our systematic review process worth noting. Due to resource constraints, we only included studies written in English. However, we contacted authors of non-English studies to obtain the English translations. In addition, although we contacted authors to share their unpublished data, only published literature was identified for inclusion. Finally, due to the numerous number of cost-effectiveness analyses included, we focused reporting on those with dominant results and a score ≥8 on the Drummond tool in the main text. We note that this is an arbitrary cut-off, and there is not an agreed upon method to provide a summary score on this tool. However, all of our results for all studies are presented in the tables and appendices despite dominance and score on the Drummond tool.

## Conclusions

We conducted a comprehensive systematic review of cost-effectiveness studies for interventions to treat adult patients with complex wounds. Our results can be used by decision-makers to assist in maximizing the deployment of clinically effective and resource efficient wound care interventions. Our analysis also highlights specific treatments that are not cost-effective, thus indicating areas for potential improvements in efficiency. A network meta-analysis and *de novo* cost-effectiveness analysis will likely bring additional clarity to the field, as some of the findings were conflicting.

## Additional files

Additional file 1:
**Wound care protocol.** Outlines the protocol used in the systematic review. Additional file 2:
**MEDLINE search strategy.** Lists MEDLINE search terms. Additional file 3:
**Classification of wound care interventions.** Lists the wound care interventions in each classification. Additional file 4:
**Drummond’s 10-item checklist tool used for cost-effectiveness analyses quality appraisal.** Provides the descriptions of the 10 items in Drummond’s 10-item checklist tool. Additional file 5:
**Cost-effectiveness analysis methodological quality appraisal results.** Lists the quality appraisal results for the 59 included cost-effectiveness analyses. Additional file 6:
**Cost-effectiveness analyses sensitivity analysis, uncertainty of results and incremental variabilities.** Outlines the sensitivity analyses, level of uncertainty, and incremental variabilities for the cost-effectiveness analyses results. Additional file 7:
**Summary of the less costly and more effective interventions for studies with a Drummond score ≥8.** Lists 42 cost-effectiveness studies with a Drummond score ≥8.

## Abbreviations

GWC: Good wound care
ICER: Incremental cost-effectiveness ratio
QALY: Quality-adjusted life-year
USD: United States dollar
