# Supplementary material for: A systematic review of cost-effectiveness analyses of complex wound interventions reveals optimal treatments for specific wound types
Source: BMC Med. 2015 Apr 22;13:90. doi: 10.1186/s12916-015-0326-3 (PMC4405871; doi:10.1186/s12916-015-0326-3)
Supplement: Additional file 1: — Wound care protocol. Outlines the protocol used in the systematic review. [file 12916_2015_326_MOESM1_ESM.pdf]

Additional file 1: Wound Care Protocol

Background
The Toronto Central Local Health Integrated Network is requesting a scoping review of best practices for wound care across the healthcare system (including primary care, acute care, rehabilitation/complex continuing care, long-term care, and community services). It will be accomplished through a scoping review of systematic reviews. The scoping review will be conducted by *breaKThrough*, a knowledge translation/synthesis center located within the Li Ka Shing Knowledge Institute of St. Michael's Hospital, directed by Dr. Sharon Straus.

Objectives
1) To conduct a systematic search to identify effective interventions for wound care across the healthcare system.
2) To characterize this literature regarding study characteristics (e.g., costing study/systematic review, number of studies identified, type of study designs included, quality), patient characteristics (e.g., clinical population, mean age), wound interventions examined, and outcomes examined.

Methods
We will use the methodologically rigorous scoping review approach proposed by previous researchers, Arksey and O'Malley. Appendix 1 displays the approach that we use to conduct a scoping review at *breaKThrough*.

Search Strategy: We will conduct systematic literature searches in the following electronic databases from inception onwards: MEDLINE (OVID interface, 1950 onwards), EMBASE (OVID interface, 1980 onwards), and the Cochrane Library (most recent issue). We will also perform targeted searches for grey literature (i.e., difficult to locate or unpublished material) by searching 1) Google and 2) the PROSPERO database of systematic reviews.

The search terms will include both medical sub-headings (MeSH) and free text termsrelated to wound care interventions. The literature search will be conducted by our experienced librarian (Laure Perrier, Librarian for the University of Toronto, Faculty of Medicine's Continuing Education and Professional Development). A preliminary search of MEDLINE and EMBASE resulted in a total of 6320 titles and abstracts (citations). We anticipate that approximately 6500 citations will be identified after searching the Cochrane Library and grey literature sources. We will further refine the search strategy iteratively with input from the investigators, individuals from Toronto Central Local Health Integrated Network, and in consultation with our experienced librarian. The search strategy will be limited to adults, systematic reviews, and economic studies, using validated search filters.

The search strategy will be peer reviewed by another librarian using the Peer Review of Electronic Search Strategies (PRESS) checklist. After this exercise, the search strategy will be amended, as required. The librarian will execute all final searches, export the results into RefWorks, and remove all duplicates from the search results. The results will then be uploaded to our Synthesi.SR tool, proprietary systematic review software of the Li Ka Shing Knowledge Institute of St. Michael's Hospital.

Inclusion criteria: The inclusion criteria will be framed using the PICOST criteria, as follows:
Patients: We will include adult patients aged 18 years and older. We anticipate that the three broad categories of patients include those with pressure ulcers, post-surgical wounds, and wounds due to chronic disease (e.g., diabetes and including arterial and venous ulcers).
Interventions: We will include all pharmacological agents used to treat wounds (e.g., topical agents), non-pharmacological agents (e.g., dressing, vacuum, honey, hyperbaric therapy), and wound care programs.
Comparators: We will include all comparators, such as other wound care interventions, no treatment, placebo, and usual care.
Outcomes: We will include healing, cost/cost-effectiveness, admission to hospital (including readmissions), and human resources.
Study designs: We will limit to systematic reviews and economic studies.
Time frame: We will not limit inclusion to year of publication.

Study Selection: Screening: Prior to commencing the screening process, a calibration exercise will be conducted to ensure reliability in correctly selecting articles for inclusion. This will entail screening a random sample of 5% of the included citations by all team members, independently. Eligibility criteria will be modified if low agreement is observed between the reviewers (e.g., a kappa statistic less than 50%). Two reviewers will then independently screen the remainder of the search results for inclusion using a pre-defined relevance criteria form for all levels of screening (e.g., title and abstract, full-text review). All levels of screening will be conducted in the SysRev Tool. Discrepancies will be resolved by discussion or the involvement of a third reviewer.

Data Abstraction: A data abstraction form will be drafted and pilot-tested by all team members independently on a random sample of 10 articles and revised iteratively by the study team while the search is completed. It is anticipated that the data items will include study characteristics (e.g., costing study/systematic review, number of studies identified, type of study designs included, quality), patient characteristics (e.g., clinical population, mean age), wound interventions examined, and outcomes examined. Two investigators will independently read each article and extract the relevant data. Data abstraction will be conducted using the SysRev Tool. Differences in abstraction will be resolved by discussion or the involvement of a third reviewer. For systematic reviews, we will appraise their quality using the Assessment of Multiple SysTemAtic Reviews (AMSTAR tool), which gives an overall score out of 11. We will not formally appraise quality fort costing studies and cost-effectiveness analysis because a validated tool does not exist.

Synthesis: Data analysis will involve quantitative (e.g., using frequency analysis) and qualitative (e.g., using thematic analysis) methods. Anticipated output from this stage of analysis will be an in-depth comparison of the literature-base, which will be depicted in a table. Contrasting wound care interventions examined in the literature will help determine the specific features required to improve care, as well as identify gaps in the literature to target future research initiatives.
