# Supplementary material for: A systematic review of cost-effectiveness analyses of complex wound interventions reveals optimal treatments for specific wound types
Source: BMC Med. 2015 Apr 22;13:90. doi: 10.1186/s12916-015-0326-3 (PMC4405871; doi:10.1186/s12916-015-0326-3)
Supplement: Additional file 2: — MEDLINE search strategy. Lists MEDLINE search terms. [file 12916_2015_326_MOESM2_ESM.pdf]

Additional file 2: MEDLINE Search Strategy

1. Pressure Ulcer/
2. (pressureadj ulcer$).tw.
3. (pressureadj sore$).tw.
4. (decubitusadj ulcer$).tw.
5. (decubitusadj sore$).tw.
6. (bedadj ulcer$).tw.
7. (bedadj sore$).tw.
8. Surgical Wound Infection/
9. Surgical Wound Dehiscence/
10. (surg$ adj2 wound$).tw.
11. (surg$ adj infect$).tw.
12. (surg$ adj2 dehiscence).tw.
13. (arterial adj2 ulcer$).tw.
14. (ischemicadj ulcer$).tw.
15. (neuropathicadj ulcer$).tw.
16. (vascul$ adj ulcer$).tw.
17. Varicose Ulcer/
18. (varicoseadj ulcer$).tw.
19. (venous adj2 ulcer$).tw.
20. (stasisadj ulcer$).tw.
21. Skin Ulcer/
22. exp Foot Ulcer/
23. (footadj ulcer$).tw.
24. Leg Ulcer/
25. (legadj ulcer$).tw.
26. (diabeticadj foot).tw.
27. (diabeticadj feet).tw.
28. (cruraladj ulcer$).tw.
29. (ulcusadjcruris).tw.
30. (chronicadj wound$).tw.
31. (chronicadj sore$).tw.
32. (chronicadj ulcer$).tw.
33. or/1-32
34. exp Adult/
35. adult.mp.
36. Middle Aged/
37. age$.tw.
38. or/34-37
39. metaanalysis.mp,pt.
40. review.pt.
41. search$.tw.
42. or/39-41 (1912330)
43. cost$.mp.
44. cost benefit analys$.mp.
45. health care costs.mp.
46. or/43-45
47. 42 or 46
48. 33 and 38 and 47
49. exp Animals/ not (exp Animals/ and Humans/)
50. 48 not 49
