# Supplementary material for: A systematic review of cost-effectiveness analyses of complex wound interventions reveals optimal treatments for specific wound types
Source: BMC Med. 2015 Apr 22;13:90. doi: 10.1186/s12916-015-0326-3 (PMC4405871; doi:10.1186/s12916-015-0326-3)
Supplement: Additional file 3: — Classification of wound care interventions. Lists the wound care interventions in each classification. [file 12916_2015_326_MOESM3_ESM.pdf]

Additional file 3: Classification of Wound Care Interventions
**1. Bandages**
2-component (outer elastic)<br>4-layer bandage<br>Compression (multi-layer, single layer, inelastic, elastic)<br>Paste bandage plus compression<br>Short stretch bandage (multi-layer, single layer)<br>Single-layer<br>High compression (elastic, multilayer)<br>No-sting barrier film<br>Unna's boot
**2. Biologics**
Arginine-glycine-aspartic acid peptide matrix (topical)<br>Autologous platelet-rich plasma/platelet-rich plasma (topical)<br>Calcitonin gene-related peptide (topical)<br>Granulocyte-macrophage colony stimulating factor (subcutaneous, peri-ulcer injection)<br>Hyaluronic acid based<br>Nerve growth factor (topical)<br>Prostaglandin E1 (intravenous)<br>Protease-modulating matrix (topical)<br>Recombinant platelet derived/fibroblast growth factor (topical)<br>Stem cell therapy<br>Sulodexide (oral)<br>Systemic mesoglycan (intra-muscular, oral)<br>Thrombin-induced human platelet growth factor (topical)<br>Thromboxane α2 antagonists (oral)<br>Transforming growth factor beta (topical)
**3. Complementary and alternative medicine**
Ayurevedic medicine (oral and topical)<br>Chinese herbal medicine (Bu-yang-huan-wu decoction, Tao-hong-si-wu decoction, Si-miao-yong-an decoction, Radix astragali, Rhizoma atractylodis, marcocephalae, Radix stephaniae tetrandrae, Radix Polygoni multifori, Radix Rehmanniae, Radix smilax china, Frusctus corni, Rhizoma dioscoreae, Cortex Moutan, Rhizoma alismatis, Rhizoma smilacis glabrae, Frutus schisandrae, Herba Siegesbeckiae, Draconis Sanguis, Lumbricus, Radix Ligustici, Chuanxiogm, Ramulus Cinnamomi Cassiae, She-xiang-huo-xue capsule)
**4. Devices/adjuvant therapy**
Electrical stimulation<br>Electromagnetic therapy<br>Hyperbaric oxygen therapy<br>Laser therapy<br>Light therapy (monochromatic, UV, polarized)<br>Magnet and normothermic therapy<br>Topical negative pressure<br>Ultrasound (high frequency, low frequency)
**5. Dressings**
Absorbent acrylic<br>Alginate<br>Aloe vera<br>Acetic acid<br>Alginate<br>Aluminin hydroxide<br>Amino acid copolymer<br>Antibiotic<br>Antiseptic agents<br>Biosynthetics<br>Bolster

Cadexomer iodine<br>Cellulose<br>Collagen<br>Copolymer membrane<br>Dextraonomer<br>Dialysate<br>Egg white<br>Eusol<br>Fibrinolysin<br>Foam<br>Honey<br>Hyaluronic acid-derived<br>Hydrocolloid<br>Hydrogel<br>Hydropolymer<br>Insulin<br>Lanolin<br>Live yeast derivative<br>Low adherent<br>Moist saline<br>Noncontact normothermic<br>Oxyquinoline<br>Papin-urea ointment<br>Paraffin gauze<br>Petrolateum<br>Phenytoin solution<br>Polyhydroxyethylmetahcrylate<br>Polyurethane<br>Polysaccharide<br>Polyurethane<br>Povidone-iodine or cadexomer-iodine<br>Radiant heat<br>Resin salve<br>Semelil gel<br>Sodium hypochlorite<br>Silicone<br>Silver<br>Streptokinase-streptodornase<br>Sugar<br>Topical enzymes<br>Transparent film<br>Vitamin A<br>Wet-to-moist gauze<br>Zinc oxide tape or salt spray
**6. Nutritional supplementation**
Arginine<br>Ascorbic acid<br>Collagen protein hydrolysate<br>Disease-specific nutrition treatment<br>High-protein diet<br>Zinc sulphate
**7. Other oral treatment**
Antibiotics (ciprofloxacin, trimethoprim, levamisole, amoxylcillin, clindamycin, oral, cefotoxin, metronidazole, gentamicin, Eusol pack, clindamycin, fluoroquinolone, rifampicin, amoxicillin/clavulanic acid, imipenem/cilastatin, cefazolin, ampicillin/sulbactam, linezolid, piperacillin/tazobactam, clindamycin hydrochloride)<br>Flavonoids

Ketanserin<br>Micronized purified flavonoid fraction (MPFF)<br>Pentoxifylline<br>Rutosides<br>Systemic ciprofloxacin<br>Zinc
**8. Other topical treatment**
Aloe vera<br>Antibiotics (polynoxilin, dimethyl sulfoxide, mupirocin)<br>Antimicrobial<br>Disinfectants<br>Enzymatic agents<br>Honey<br>Honey<br>Hydrogel<br>Ketanserin<br>Ketanserin ointment (2%) (topical)<br>Lyophilized collagen<br>Mesoglycan<br>Phenytoin<br>Plant-based extract<br>Polyethylene glycol<br>Procaine<br>Silver<br>Topical sugar
**9. Skin replacement therapy**
Allografts (fresh, split thickness)<br>Applied freeze-dried keratinocyte lysate<br>Cryopreserved allografts<br>Cultured keratinocytes/epidermal/ allogenic bilayer<br>Fibroblast-derived<br>Split thickness graft<br>Tissue engineered skin
**10. Stockings**
Casting (Total contact casting, plaster cast)<br>Compression<br>High-compression regimens<br>Intermittent pneumatic compression<br>Multilayer elastic system, multilayer elastomeric (or non-elastomeric)<br>Single-layer non-elastic system
**11. Support surfaces**
Air-fluidised bed/supports<br>Air mattress<br>Alternating pressure mattress<br>Fluid mattress overlay<br>Foam mattress (alternative foam, specialized foam)<br>Low air-loss beds<br>Low-tech constant-low-pressure supports<br>Pressure off-loading (pressure-relief half shoe)<br>Pressure off-loading (total contact or non-removable cast)<br>Pressure off-loading (felted foam)<br>Seat cushions<br>Therapeutic footwear<br>Water mattress
**12. Surgery**

Early surgical intervention<br>Endovascular or open bypass revascularization surgery of an ulcerated foot<br>Percutaneous flexor tenotomy<br>Pinch grafting<br>Resection of the chronic wound<br>Subfascial endoscopic perforator surgery<br>Superficial vein surgery<br>Superficial venous surgery
**13. Wound care programs**
Foot care clinic<br>Leg ulcer clinics<br>Patient education<br>Primary nurse delivery, clinician education, standardized treatment, team of community pharmacists and nurses
**14. Wound cleansing**
Cadexomer iodine<br>Cadexomer iodine<br>Collagenase debridement (topical)<br>Larval therapy<br>Wound cleansing agents (collagenase, topical)<br>Wound cleansing (Maggot debridement therapy)<br>Wound cleansing agents (Dextranomer polysaccharide beads or paste, Cadexomer iodine polysaccharide beads or paste)<br>Wound cleansing agents (Dextranomer polysaccharide beads or paste, Cadexomer iodine polysaccharide beads or paste)<br>Wound cleansing agents (Dextranomer polysaccharide beads or paste, Cadexomer iodine polysaccharide beads or paste)<br>Larval Therapy<br>Topical agents (superoxidized water and soap, povidone iodine)<br>Wound cleansing (Sharp debridement)<br>Wound cleansing (saline spray containing aloe vera, silver chloride and decylglucoside (Vulnopur), saline, whirlpool)<br>Wound cleansing (collagenase enzymatic)<br>Wound cleansing (collagenase)
