# Supplementary material for: A systematic review of cost-effectiveness analyses of complex wound interventions reveals optimal treatments for specific wound types
Source: BMC Med. 2015 Apr 22;13:90. doi: 10.1186/s12916-015-0326-3 (PMC4405871; doi:10.1186/s12916-015-0326-3)
Supplement: Additional file 4: — Drummond’s 10-item checklist tool used for cost-effectiveness analyses quality appraisal. Provides the descriptions of the 10 items in Drummond’s 10-item checklist tool. [file 12916_2015_326_MOESM4_ESM.pdf]

Additional file 4: Drummond's 10-item Checklist Tool[a] Used for CEA Quality Appraisal

Excel Column	Description
**Q1. Was a well-defined question posed in answerable form?**	1.1. Did the study examine both costs and effects of the service(s) or programme(s)?<br>1.2. Did the study involve a comparison of alternatives?<br>1.3. Was a viewpoint for the analysis stated and was the study placed in any particular decision-making context?
**Q2. Was a comprehensive description of the competing alternatives given (i.e. can you tell who did what to whom, where, and how often)?**	2.1. Were any relevant alternatives omitted?<br>2.2. Was (should) a do-nothing alternative (be) considered?
**Q3. Was the effectiveness of the programme or services established?**	3.1. Was this done through a randomised, controlled clinical trial? If so, did the trial protocol reflect what would happen in regular practice?<br>3.2. Were effectiveness data collected and summarized through a systematic overview of clinical studies? If so, were the search strategy and rules for inclusion or exclusion outlined?<br>3.3. Were observational data or assumptions used to establish effectiveness? If so, what are the potential biases in results?
**Q4. Were all the important and relevant costs and consequences for each alternative identified?**	4.1. Was the range wide enough for the research question at hand?<br>4.2. Did it cover all relevant viewpoints? (Possible viewpoints include the community or social viewpoint, and those of patients and third-party payers. Other viewpoints may also be relevant depending upon the particular analysis.)<br>4.3. Were the capital costs, as well as operating costs, included?
**Q5. Were costs and consequences measured accurately in appropriate physical units (for example, hours of nursing time, number of physician visits, lost work-days, gained life-years)?**	5.1. Were the sources of resource utilization described and justified?<br>5.2. Were any of the identified items omitted from measurement? If so, does this mean that they carried no weight in the subsequent analysis?<br>5.3. Were there any special circumstances (e.g., joint use of resources) that made measurement difficult? Were these circumstances handled appropriately?
**Q6. Were costs and consequences valued credibly?**	6.1. Were the sources of all values clearly identified? (Possible sources include market values, patient or client preferences and views, policy-makers' views, and health professionals' judgements)<br>6.2. Were market values employed for changes involving resources gained or depleted?<br>6.3. Where market values were absent (e.g. volunteer labour), or market values did not reflect actual values (such as clinic space donated at a reduced rate), were adjustments made to approximate market values?<br>6.4. Was the valuation of consequences appropriate for the question posed (i.e. has the appropriate type or types of analysis – cost-effectiveness, cost-benefit, cost-utility – been selected)?
**Q7. Were costs and consequences adjusted for differential timing?**	7.1. Were costs and consequences that occur in the future 'discounted' to their present values?<br>7.2. Was any justification given for the discount rate used?
**Q8. Was an incremental analysis of costs and consequences of alternatives**	8.1. Were the additional (incremental) costs generated by one alternative over another compared to the additional effects, benefits, or utilities generated?

**performed?**	
**Q9. Was allowance made for uncertainty in the estimates of costs and consequences?**	9.1. If patient-level data on costs or consequences were available, were appropriate statistical analyses performed?<br>9.2. If a sensitivity analysis was employed, was justification provided for the ranges or distributions of values (for key study parameters), and the form of sensitivity analysis used?<br>9.3. Were the conclusions of the study sensitive to the uncertainty in the results, as quantified by the statistical and/or sensitivity analysis?
**Q10. Did the presentation and discussion of study results include all issues of concern to users?**	10.1. Were the conclusions of the analysis based on some overall index or ratio of costs to consequences (for example, cost-effectiveness ratio)? If so, was the index interpreted intelligently or in a mechanistic fashion?<br>10.2. Were the results compared with those of others who have investigated the same question? If so, were allowances made for potential differences in study methodology?<br>10.3. Did the study discuss the generalizability of the results to other settings and patient/client groups?<br>10.4. Did the study allude to, or take account of, other important factors in the choice or decision under consideration (for example, distribution of costs and consequences, or relevant ethical issues)?<br>10.5. Did the study discuss issues of implementation, such as the feasibility of adopting the 'preferred' programme given existing financial or other constraints, and whether any freed resources could be redeployed to other worthwhile programmes?

[a]Drummond MF, Drummond MF. *Methods for the economic evaluation of health care programmes.* 3rd ed. Oxford ; New York: Oxford University Press; 2005.
