# Supplementary material for: A systematic review of cost-effectiveness analyses of complex wound interventions reveals optimal treatments for specific wound types
Source: BMC Med. 2015 Apr 22;13:90. doi: 10.1186/s12916-015-0326-3 (PMC4405871; doi:10.1186/s12916-015-0326-3)
Supplement: Additional file 5: — Cost-effectiveness analysis methodological quality appraisal results. Lists the quality appraisal results for the 59 included cost-effectiveness analyses. [file 12916_2015_326_MOESM5_ESM.pdf]

Additional file 5: CEA Methodological Quality Appraisal Results

Study	Q1	Q2	Q3	Q4	Q5	Q6	Q7	Q8	Q9	Q10	Total Drummond Score
Abidia, 2003	Y	Y	N	Y	Y	Y	NA	N	N	N	5
Apelqvist, 1996	Y	Y	N	Y	Y	Y	NA	Y	Y	Y	8
Augustin, 1999	Y	Y	N	Y	Y	Y	NA	N	N	Y	6
Bale, 1998	Y	Y	Y	Y	Y	Y	NA	N	Y	Y	8
Branom, 2001	Y	Y	N	Y	Y	Y	NA	N	N	N	5
Burgos, 2000	Y	Y	N	Y	Y	Y	NA	Y	Y	N	7
Chang, 1998	Y	Y	N	Y	Y	Y	NA	Y	Y	N	7
Chuangsuwanich, 2011	Y	Y	N	Y	Y	Y	NA	Y	Y	N	7
DePalma, 1999	Y	Y	N	Y	Y	Y	NA	Y	Y	N	7
Dumville, 2009	Y	Y	Y	Y	Y	Y	NA	Y	Y	Y	9
Edmonds, 1999	Y	Y	N	Y	Y	Y	NA	Y	Y	Y	8
Ferrell, 1995	Y	Y	N	Y	Y	Y	NA	Y	Y	Y	8
Foglia, 2012	Y	Y	Y	N	Y	Y	NA	Y	Y	Y	8
Glinski, 1999	Y	Y	Y	Y	Y	Y	NA	Y	N	Y	8
Gordon, 2006	Y	Y	N	Y	Y	Y	NA	Y	Y	Y	8
Graumlich, 2003	Y	Y	N	Y	Y	Y	NA	Y	Y	Y	8
Guest, 2012	Y	Y	Y	Y	Y	Y	NA	Y	Y	Y	9
Guo, 2003	Y	Y	N	Y	Y	Y	Y	Y	Y	Y	9
Habacher, 2007	Y	Y	N	Y	Y	Y	Y	Y	Y	Y	9
Horswell, 2003	Y	Y	Y	Y	Y	Y	NA	Y	Y	Y	9
Iglesias, 2006	Y	Y	Y	Y	Y	Y	Y	Y	Y	Y	10
Iglesias, 2004	Y	Y	Y	Y	Y	Y	Y	Y	Y	Y	10
Jansen, 2009	Y	Y	Y	Y	Y	Y	Y	Y	Y	Y	10
Jeffcoate, 2009	Y	Y	Y	Y	Y	Y	NA	Y	Y	Y	9
Jull, 2008	Y	Y	Y	Y	Y	Y	NA	Y	Y	Y	9
Junger, 2008	Y	Y	N	Y	N	Y	NA	N	Y	N	5
Kerstein, 2000	Y	Y	N	Y	N	Y	N	N	N	N	4
Kikta, 1988	Y	Y	Y	Y	Y	Y	NA	Y	Y	Y	9
McKinNn, 1997	Y	Y	Y	Y	Y	Y	NA	Y	Y	Y	9
Michaels, 2009	Y	Y	Y	Y	Y	Y	NA	Y	Y	Y	9
Morrell, 1998	Y	Y	Y	Y	Y	Y	NA	Y	Y	Y	9
Muller, 2001	Y	Y	N	Y	Y	Y	NA	Y	Y	Y	8
Narayanan, 2005	Y	Y	N	Y	Y	Y	NA	N	N	N	5
O'Brien, 2003	Y	Y	Y	Y	Y	Y	NA	Y	Y	Y	9
Ohlsson, 1994	Y	Y	N	Y	Y	Y	NA	Y	Y	Y	8
Oien, 2001	Y	Y	N	Y	Y	Y	NA	N	Y	Y	7
Payne, 2009	Y	Y	N	Y	Y	Y	NA	Y	Y	N	7

Persson, 2000	Y	Y	Y	Y	Y	Y	Y	Y	Y	Y	10
Piaggesi, 2007	Y	Y	N	N	Y	Y	NA	Y	Y	Y	7
Robson, 2000	Y	Y	N	Y	Y	Y	NA	N	N	N	5
Sanada, 2010	Y	Y	N	Y	Y	Y	NA	N	Y	Y	7
Sibbald, 2001	Y	Y	Y	Y	Y	Y	NA	Y	Y	Y	9
Taylor, 1998	Y	Y	N	Y	Y	Y	NA	Y	Y	Y	8
Terry, 2009	Y	Y	N	Y	Y	Y	NA	N	Y	Y	7
Ukat, 2003	Y	Y	Y	Y	Y	Y	NA	Y	N	Y	8
Vu, 2007	Y	Y	Y	Y	Y	Y	NA	Y	Y	Y	9
Watson, 2011	Y	Y	Y	Y	Y	Y	Y	Y	Y	Y	10
Xakellis, 1992	Y	Y	N	Y	Y	Y	NA	Y	Y	Y	8
Pham, 2012	Y	Y	Y	Y	Y	Y	Y	Y	Y	Y	10
Redekop, 2003	Y	Y	Y	Y	Y	Y	NA	Y	Y	Y	9
Schonfeld, 2000	Y	Y	Y	Y	Y	Y	NA	Y	Y	Y	9
Allenet, 2000	Y	Y	Y	Y	Y	Y	NA	Y	Y	Y	9
Simon, 1996	Y	Y	N	Y	Y	Y	NA	N	N	N	5
Carr, 1999	Y	Y	Y	Y	Y	Y	NA	Y	Y	Y	9
Ghatnekar, 2002	Y	Y	Y	Y	Y	Y	Y	Y	Y	Y	10
Guest, 2009	Y	Y	Y	Y	Y	Y	NA	Y	Y	Y	9
Sebern, 1986	Y	Y	N	Y	Y	Y	NA	Y	Y	Y	8
Ghatnekar, 2001	Y	Y	Y	Y	Y	Y	NA	Y	Y	Y	9
Hailey, 2007	Y	Y	Y	Y	Y	Y	Y	Y	Y	Y	10

Abbreviations: CEA=cost-effectiveness analysis, NA=not applicable, N=no, Q=question, Y=yes.
