# Supplementary material for: A systematic review of cost-effectiveness analyses of complex wound interventions reveals optimal treatments for specific wound types
Source: BMC Med. 2015 Apr 22;13:90. doi: 10.1186/s12916-015-0326-3 (PMC4405871; doi:10.1186/s12916-015-0326-3)
Supplement: Additional file 6: — Cost-effectiveness analyses sensitivity analysis, uncertainty of results and incremental variabilities. Outlines the sensitivity analyses, level of uncertainty, and incremental variabilities for the cost-effectiveness analyses results. [file 12916_2015_326_MOESM6_ESM.pdf]

Additional file 6: CEA Sensitivity Analyses, Uncertainty of Results and Incremental Variabilities

CEA (Original Yr of Values)	Intervention vs. Comparator	Sensitivity Analyses[a]	Uncertainty of Result[b] and Incremental Variabilities[c]
VENOUS ULCERS (n=24)			
Augustin 1999 (1989)	hydrocolloid dressing vs. Vaseline gauze dressing	NR	U: NR.<br>VC: NR;<br>VE: not statistically significant
DePalma 1999 (1998)	Thera-boot vs. Unna's boot	NR	U: NR.<br>VC: p=0.05;<br>VE: p=0.41
Glinski 1999 (1998)	micronised purified flavonoid fraction+SC vs. SC alone	NR	U: NR.<br>VC: NR;<br>VE: p<0.05
Gordon 2006 (2005)	community Leg Club vs. community home nursing	healing rate, bandage & dressing expenses, volunteer in-kind support	U: NR.<br>VC: NR;<br>VE: statistically significant
Guest 2012 (2010)	No Sting Barrier Film (NSBF) vs. Durable Barrier Cream (DBC)	number of nurse visits, healing rate	U: BST; Distribution in costs and QALYs is the same for NSBF and control groups; distribution of costs in DBC group is marginally less than the other two groups.
Guest 2012 (2010)	NSBF vs. no skin protectant	number of nurse visits, healing rate	U: BST; Distribution in costs and QALYs is the same for NSBF and control groups; distribution of costs in DBC group is marginally less than the other two groups.
Guest 2012 (2010)	DBC vs. no skin protectant	number of nurse visits, healing rate	U: BST; Distribution in costs and QALYs is the same for NSBF and control groups; distribution of costs in DBC group is marginally less than the other two groups.
Iglesias 2006 (2004)	pentoxifylline plus compression vs. placebo plus compression	nil.	U: CEAC; Probabilities of pentoxifylline being cost-effective at WTP values of £0, £500 and £1000 per QALY gained were 0.90, 0.91 and 0.89, respectively.
Iglesias 2004 (2001)	four-layer bandage vs. short-stretch bandage	nil.	U: BST; Vast majority of point estimates fall in the 'Dominant' quadrant.
Jull 2008 (2005)	manuka honey dressing vs. UC	NR	U[‡]: 95% CI of ICER: (-3396, 1377).
Junger 2008 (2007)	low-frequency pulsed current (Dermapulse) vs. placebo	nil.	U: NR.<br>VC: not statistically significant;<br>VE: not statistically significant
Kerstein 2000 (1995)	hydrocolloid dressing+compression hosiery vs. Unna's boot	NR	NR
Kerstein 2000 (1995)	Unna's boot vs. saline gauze	NR	NR
Kikta 1988	Unna's boot vs. hydrocolloid (DuoDERM)	NR	U: NR.<br>VC: not statistically significant;

(1987)			VE: p=0.01
Michaels 2009 (2007)	antimicrobial silver-donating dressings vs. low-adherent dressings	nil.	U: BST; 30% of the replications located in the NE quadrant (more costly, more effective); 46% located in the NW quadrant (antimicrobial dressings dominated by control dressings).
Morrell 1998 (1995)	community leg ulcer clinics using four-layer compression bandaging vs. home nursing UC	nil.	U: 95% CI of ICER: (-96, 298).
O'Brien 2003 (2000)	four-layer bandage vs. UC	NR	U: NR.<br>VC: statistically significant;<br>VE: p=0.015
Oien 2001 (1997)	pinch grafting in primary care vs. pinch grafting in hospital	NR	U: NR.<br>VC: p<0.001<br>VE: NA
Sibbald 2001 (1997)	skin substitute (Apligraf) plus four-layer bandage vs. four-layer bandage only	time loss from usual daily activities	NR
Taylor 1998 (1987)	four-layer high-compression bandaging vs. UC	NR	U: NR.<br>VC: p=0.016;<br>VE: p=0.003
Ukat 2003 (2002)	multilayer elastic bandaging (Profore) vs. short-stretch bandaging	NR	U: NR.<br>VC: NR;<br>VE: statistically significant
Watson 2011 (2007)	ultrasound plus SC vs. SC alone	nil.	U: BST; 67% of points fall in the 'Dominated' quadrant.
Pham 2012 (2009)	four-layer bandaging (4LB) vs. short-stretch bandaging (SSB)	nil.	U: CEAC; For willingness-to-pay values between CAN$50,000 and CAN$100,000 per QALY, the probability that 4LB is more cost-effective than SSB ranged from 51% to 63%.
Schon-feld 2000 (1996)	Apligraf (Graftskin) vs. Unna's Boot	nil.	NR
Simon 1996 (1993)	community leg ulcer clinic vs. UC clinic	NR	NR
Carr 1999 (1998)	four-layer compression bandaging (Profore) vs. UC	nil.	NR
Guest 2009 (2007)	Amelogenin plus compression therapy vs. compression therapy only	probability of healing	U: BST; Majority of points (not %) fall in the 'Dominant' quadrant.
VENOUS & VENOUS/ARTERIAL ULCERS (n=2)			
Dumville 2009 (2006)	larval therapy vs. hydrogel	nil.	U: BST; Points fall in all quadrants fairly symmetrically, suggesting high levels of uncertainty.
Ohlsson 1994 (1993)	hydrocolloid (DuoDERM) dressing vs. saline gauze	NR	U: NR.<br>VC: p<0.009;<br>VE: not statistically significant
DIABETIC ULCERS (n=16)			
Abidia	hyperbaric oxygen therapy	NR	U: NR.

2003 (2000)	(HBOT) vs. control		VC: NR;<br>VE: p=0.026
Apelqvist 1996 (1993)	cadexomer iodine ointment vs. standard treatment	hospitalization	U: NR.<br>VC: p<0.001;<br>VE: not statistically significant
Edmonds 1999 (1996)	Filgrastim vs. placebo	nil.	U: NR<br>VC: NR;<br>VE: p=0.02
Guo 2003 (2001)	HBOT+SC vs. SC alone	efficaciousness probabilities, quality weights, number of HBOT treatments, HBOT cost per treatment, treatment costs of major and minor amputations per case	NR
Habacher 2007 (2001)	intensified treatment vs. SC	re-ulceration rate	NR
Horswell 2003 (1999)	Staged Management Diabetes Foot Program vs. SC	NR	U: NR.<br>VC: p=0.014;<br>VE: p=0.0002
Jansen 2009 (2006)	ErtapenemvsPiperacillin/Tazobactam	initial antimicrobial resistance	U: BST; Vast majority of the distribution falls in the 'Dominant' quadrant (exact % NR).
Jeffcoate 2009 (2007)	hydrocolloid (Aquacel) vs. antiseptic (Inadine)	changes in costs	U: NR.<br>VC: not statistically significant;<br>VE: not statistically significant
Jeffcoate 2009 (2007)	antiseptic (Inadine) vs. non-adherent dressing	changes in costs	U: NR.<br>VC: not statistically significant;<br>VE: p=0.39
McKin-non 1997 (1994)	ampicillin/sulbactamvsimipenem/cilastatin	clinical success rate for each drug	U: NR.<br>VC: NR;<br>VE: not statistically significant
Persson 2000 (1999)	becaplermin plus good wound care (GWC) vs. GWC alone	healing rate	NR
Piaggesi 2007 (2006)	total contact casting vs. Optima Diab device	NR	U: NR.<br>VC: p<0.001;<br>VE: not statistically significant
Redekop 2003 (1999)	Apligraf (skin substitute)+GWC vs. GWC alone	number of applications of Apligraf required, amputation rate and costs, infection rate, healing rate	NR
Allenet 2000 (1998)	Dermagraft (human dermal replacement) vs. SC	nil.	NR
Ghatnekar 2002 (2000)	Promogran dressing plus GWC vs. GWC alone	healing rate, number of dressing changes	U: NR.<br>VC: NR;<br>VE: not statistically significant
Ghatne-kar 2001 (1999)	becaplermin gel (containing recombinant human platelet-derived growth factor) plus GWC vs. GWC alone	healing rate, patient’s prospects for healing and worsening	NR
Hailey 2007	HBOT+SC vs. SC alone	nil.	NR

(2004)			
PRESSURE ULCERS (n=14)			
Branom 2001 (2000)	Constant Force Technology mattress vs. low-air-loss mattress	NR	NR
Burgos 2000 (1998)	collagenase ointment vs. hydrocolloid (Varihesive) dressing	NR	U: NR.<br>VC: not statistically significant;<br>VE: not statistically significant
Chang 1998 (1997)	hydrocolloid (DuoDERM CGF) vs. saline gauze	NR	U: NR.<br>VC: not statistically significant;<br>VE: not statistically significant
Chuang-suwanich 2011 (2010)	silver mesh dressing vs. silver sulfadiazine cream	NR	U: NR.<br>VC: p=0.0001;<br>VE: not statistically significant
Ferrell 1995 (1992)	low-air-loss bed vs. conventional foam mattress	low-air-loss bed lease cost, patient & wound healing characteristics	NR
Foglia 2012 (2010)	advanced dressings vs. simple dressings	nil.	U: NR.<br>VC: NR;<br>VE: p=0.05
Graum-lich 2003 (2001)	collagen vs. hydrocolloid (DuoDERM)	nil.	U: NR.<br>VC: NR;<br>VE: p=0.89
Muller 2001 (1998)	collagenase-containing ointment vs. hydrocolloid (DuoDERM) dressing	nil.	U: NR.<br>VC: NR;<br>VE: p<0.005
Naraya-nan 2005 (2004)	Initial wound stage 1: BCT (balsam Peru+hydrogenated castor oil+trypsin ointment) only vsBCT+Others (BCT plus Other treatments)	NR	U: NR.<br>VC: NR;<br>VE: not statistically significant
Naraya-nan 2005 (2004)	Initi<br>al wound stage 1: BCT+Others vs. Others	NR	U: NR.<br>VC: NR;<br>VE: not statistically significant
Naraya-nan 2005 (2004)	Initial wound stage 2: BCT only vs. Others	NR	U: NR.<br>VC: not statistically significant;<br>VE: not statistically significant
Naraya-nan 2005 (2004)	Initial wound stage 2: BCT only vs. BCT+Others	NR	U: NR.<br>VC: NR;<br>VE: not statistically significant
Naraya-nan 2005 (2004)	Initial wound stage 2: BCT+Others vs. Others	NR	U: NR.<br>VC: NR;<br>VE: not statistically significant
Payne 2009 (2007)	polyurethane foam dressing (Allevyn Thin) vs. saline gauze	material costs, frequency of dressing changes	U: NR.<br>VC: p=0.055;<br>VE: NR
Robson 2000 (1999)	sequential granulocyte-macrophage/colony-stimulating factor (GM-CSF) and basic fibroblast growth factor (bFGF) vs. bFGF only	NR	U: NR.<br>VC: NR;<br>VE: not statistically significant
Robson 2000 (1999)	sequential GM-CSF and bFGF vs. GM-CSF only	NR	U: NR.<br>VC: NR;<br>VE: not statistically significant

Robson 2000 (1999)	placebo vs. sequential GM-CSF and bFGF	NR	U: NR.<br>VC: NR;<br>VE: not statistically significant
Sanada 2010 (2007)	new incentive system vs. non-introduced control	NR	U: NR.<br>VC: not statistically significant;<br>VE: p<0.001
Xakellis 1992 (1990)	hydrocolloid (DuoDERM) vs. gauze	NR	U: NR.<br>VC: p=0.04;<br>VE: p=0.12
Sebern 1986 (1985)	Grade II PrU: moisture vapor permeable dressing (MVP) vs. gauze	NR	U: NR.<br>VC: p<0.05;<br>VE: p<0.01
Sebern 1986 (1985)	Grade III PrU: MVP vs. gauze	NR	U: NR.<br>VC: not statistically significant;<br>VE: not statistically significant
MIXED WOUNDS (n=3)			
Bale 1998 (1994)	hydrocellular (Allevyn) dressing vs. hydrocolloid (Granuflex) dressing	NR	U: NR.<br>VC: NR;<br>VE: p=0.045
Terry 2009 (2008)	telemedicine plus wound care specialist (WCS) consults vs. WCS consults only	NR	U: NR.<br>VC: statistically significant;<br>VE: NR
Vu 2007 (2000)	multidisciplinary wound care team vs. UC	nil.	U: CEAC; The intervention resulted in both significant cost savings and significantly improved outcomes.

Abbreviations: BCT=balsam Peru plus hydrogenated castor oil plus trypsin ointment; bFGF=basic fibroblast growth factor; CEA=cost-effectiveness analysis; CEAC=cost-effectiveness acceptability curve; DBC=Durable Barrier Cream; ICER=incremental cost-effectiveness ratio; GM-CSF=granulocyte-macrophage/colony-stimulating factor; GWC=good wound care; HBOT=hyperbaric oxygen therapy; MVP= moisture vapor permeable dressing; NR=not reported; NSBF=No Sting Barrier Film; PrU=pressure ulcer; QALY=quality-adjusted life-year; SC=standard care; UC=usual care; US$=United States dollars; WCS= wound care specialist; WTP= willingness-to-pay; Yr=year; Vs.=versus.

Notes:

[a]Only variables that changed the classification of results are noted here (i.e., changed cost-effectiveness quadrants); nil=the sensitivity analysis variables did not change the classification of results (i.e., did not change cost-effectiveness quadrants); NR=not reported/conducted.

[b]U=Uncertainty of the CE result; including from Bootstrapping (BST), Cost-effectiveness acceptability curve (CEAC) or 95% CI of the ICER.

[c]VC=Variability of incremental cost; & VE=Variability of incremental effectiveness; including p-value, 95% CI or statistical significance.
