# Supplementary material for: A systematic review of cost-effectiveness analyses of complex wound interventions reveals optimal treatments for specific wound types
Source: BMC Med. 2015 Apr 22;13:90. doi: 10.1186/s12916-015-0326-3 (PMC4405871; doi:10.1186/s12916-015-0326-3)
Supplement: Additional file 7: — Summary of the less costly and more effective interventions for studies with a Drummond score ≥8. Lists 42 cost-effectiveness studies with a Drummond score ≥8. [file 12916_2015_326_MOESM7_ESM.pdf]

Additional file 7: Summary of the Less Costly and More Effective Interventions for Studies with a Drummond Score ≥8

Wound type	Treatment comparisons (n=22)		
Venous ulcers	Four-layer compression bandaging	vs.	Usual care
	Apligraf (Graftskin)	vs.	Unna's Boot
	Unna's boot	vs.	Hydrocolloid (DuoDERM)
	Micronised purified flavonoid fraction plus standard care	vs.	Standard care
	Durable Barrier Cream	vs.	No skin protectant
	Pentoxifylline plus compression	vs.	Placebo plus compression
	Manuka honey dressing	vs.	Usual care
	Amelogenin plus compression therapy	vs.	Compression therapy only
Mixed venous and venous/arterial ulcers	Hydrocolloid (DuoDERM) dressing	vs.	Saline gauze
Diabetic ulcers	Cadexomer iodine ointment	vs.	Standard treatment
	Filgrastim	vs.	Placebo
	Intensified treatment	vs.	Standard care
	Staged Management Diabetes Foot Program	vs.	Standard care
	Ertapenem	vs.	Piperacillin/Tazobactam
	Ampicillin/sulbactam	vs.	imipenem/cilastatin
	Becaplermin plus good wound care	vs.	Good wound care alone
	Apligraf (skin substitute) plus good wound care	vs.	Good wound care alone
	Promogran dressing plus good wound care	vs.	Good wound care alone
Pressure ulcers	Moisture vapor permeable dressing	vs.	Gauze
	Advanced dressings	vs.	simple dressings
	Hydrocolloid (DuoDERM)	vs.	gauze
Mixed wound types	multidisciplinary wound care team	vs.	Usual care
